# Comparative Genomics of Neuroglobin Reveals Its Early Origins

**DOI:** 10.1371/journal.pone.0047972

**Published:** 2012-10-25

**Authors:** Jasmin Dröge, Amit Pande, Ella W. Englander, Wojciech Makałowski

**Affiliations:** 1 Institute of Bioinformatics, Faculty of Medicine, University of Muenster, Muenster, Germany; 2 Department of Surgery, University of Texas Medical Branch, Galveston, Texas, United States of America; Ecole Normale Supérieure de Lyon, France

## Abstract

**Background:**

Neuroglobin (Ngb) is a hexacoordinated globin expressed mainly in the central and peripheral nervous system of vertebrates. Although several hypotheses have been put forward regarding the role of neuroglobin, its definite function remains uncertain. Ngb appears to have a neuro-protective role enhancing cell viability under hypoxia and other types of oxidative stress. Ngb is phylogenetically ancient and has a substitution rate nearly four times lower than that of other vertebrate globins, e.g. hemoglobin. Despite its high sequence conservation among vertebrates Ngb seems to be elusive in invertebrates.

**Principal Findings:**

We determined candidate orthologs in invertebrates and identified a globin of the placozoan *Trichoplax adhaerens* that is most likely orthologous to vertebrate Ngb and confirmed the orthologous relationship of the polymeric globin of the sea urchin *Strongylocentrotus purpuratus* to Ngb. The putative orthologous globin genes are located next to genes orthologous to vertebrate *POMT2* similarly to localization of vertebrate Ngb. The shared syntenic position of the globins from *Trichoplax*, the sea urchin and of vertebrate Ngb strongly suggests that they are orthologous. A search for conserved transcription factor binding sites (TFBSs) in the promoter regions of the Ngb genes of different vertebrates via phylogenetic footprinting revealed several TFBSs, which may contribute to the specific expression of Ngb, whereas a comparative analysis with myoglobin revealed several common TFBSs, suggestive of regulatory mechanisms common to globin genes.

**Significance:**

Identification of the placozoan and echinoderm genes orthologous to vertebrate neuroglobin strongly supports the hypothesis of the early evolutionary origin of this globin, as it shows that neuroglobin was already present in the placozoan-bilaterian last common ancestor. Computational determination of the transcription factor binding sites repertoire provides on the one hand a set of transcriptional factors that are responsible for the specific expression of the Ngb genes and on the other hand a set of factors potentially controlling expression of a couple of different globin genes.

## Introduction

Globins are small heme proteins that bind various external ligands, such as oxygen or nitric oxide, and they are found in all kingdoms of life [1], [2], [3]. Vertebrate genomes harbor seven different globin types, including prominent hemoglobin (Hb) and myoglobin (Mb). Tetrameric Hb is present in erythrocytes and transports oxygen via blood to the inner tissues [4]. The monomeric, muscle-specific myoglobin, on the other hand, stores oxygen, enhances the oxygen diffusion to the muscle cells and detoxifies nitric oxide [5]. Five recent additions to vertebrate globin repertoire are cytoglobin (Cygb), neuroglobin (Ngb), globin X (GbX), globin E (GbE) and globin Y (GbY). GbE is an eye-specific globin protein exclusively found in avian species [6], [7]. GbY is only present in amphibians, reptiles and monotreme mammals in contrast to GbX which is a membrane-bound globin specific to amphibians, reptiles, the sea lamprey and fishes [8], [9], [10], [11], [12]. It has been suggested that the GbE and Mb genes, as well as the GbY and proto-Hb genes, are the paralogous products of tandem gene duplications that occurred early in the evolution of vertebrates [7], [10], [13], [14], [15]. Phylogenetic and genomic analyses indicate that the Cygb, Hb/GbY and Mb/GbE genes are products of two rounds of whole-genome duplications in the stem lineage of vertebrates [14], [15]. In contrast, Ngb and GbX derive from independent duplication events that occurred before the split of Deuterostomia and Protostomia [11], [14], [16]. To date little is known regarding the functions of the novel globin proteins, except of Cygb and Ngb which have been extensively studied in recent years. Cygb is a dimeric protein that is expressed in fibroblast-like cells and in a distinct population of neurons [17], [18], [19], [20], [21] and it is upregulated under hypoxic conditions [17], [22], [23], [24], [25]. Ngb is mainly expressed in the nervous system and to lesser extend in some endocrine tissues, like the adrenal and pituitary glands [16], [26], [27], [28], [29].

The overall expression of Ngb in the mouse brain is very low. In contrast, its concentration in retinal neurons appears to be significantly higher and comparable to the concentration of Mb in muscles [30]. Like Mb, Ngb is a monomeric protein that features the typical 3/3 α-helical globin fold [31]. The main structural difference between Mb and Ngb is the coordination of the iron atom in the deoxy state [32]. In Mb and Hb the central iron atom is pentacoordinated in the deoxy state and external ligands can bind to the free sixth coordination site. In Ngb, and also in Cygb, the sixth coordination site is occupied by the distal histidine of the E helix (HisE7). Thus, this internal ligand has to be displaced before any external ligand can bind to the iron atom [16], [32], [33], [34]. Although the function of Ngb has been widely discussed, its exact physiological role is still unknown [35]. However, it has been suggested that Ngb protects neurons from hypoxia-related stress [36], [37], [38] and from amyloid-induced neurotoxicity [39]. Several functions have been proposed. The most parsimonious hypothesis seems to be an oxygen delivery function of Ngb akin to invertebrates’ nerve globins, which are involved in oxygen supply [16], [32]. It has been suggested that Ngb may neutralize damaging reactive oxygen or nitrogen species, detoxify harmful nitric oxide to nitrate [40], [41], or act as redox regulated nitrite reductase [42]. Moreover, it may be involved in a signal transduction pathway by inhibiting the dissociation of GDP from G protein α [43] and in a redox reaction to prevent apoptosis via reduction of cytochrome c [44]. Till now, Ngb orthologs have been identified in all examined gnathostomes. Ngb seems to be a single-copy gene, with the only known exception being the rainbow trout (*Oncorhynchus mykiss*) genome, which possesses two distinct Ngb genes [45]. Ngb is highly conserved from mammals to fish showing ∼ 55% identities and up to 80% similarities [16], [46]. The very strong purifying selection points to an important function of this protein [45]. Interestingly, the nerve globin of the annelid *Aphrodite aculeata* is more similar to Ngb (30% amino acid identity) than vertebrate Mb and Hb, with 21% and 25% amino acid sequence identity, respectively [16]. This led to the conclusion that Ngbs belong to an ancient globin family that originated early in the evolution of the metazoan [16]. Thus, the question arises, did Ngb first appear with the emergence of the nervous system and evolved concurrently with it? The close relationship to annelid intracellular globins, e.g. the nerve globin of *A. aculeate*, indicates a wide distribution of Ngb. Moreover, only recently a globin protein orthologous to neuroglobin has been identified in the cephalochordate *Branchiostoma floridae* [47]. Here we present a comparative study revolving around Ngb biology in a large sense with focus on those aspects of Ngb that can be studied computationally. Our phylogenetic analysis led to the identification of orthologs of Ngb in the placozoan *Trichoplax adhaerens* and in the sea urchin *Strongylocentrotus purpuratus*. Additionally, we were able to identify several conserved putative transcription factor-binding sites via phylogenetic footprinting that may contribute to the specific expression of Ngb in the central and peripheral nervous system of vertebrates.

## Results and Discussion

Neuroglobin, globin X, the hemoglobins from *Ciona*, as well as some invertebrate nerve and intracellular globins, belong to an ancient branch of globins that diverged from other globin types before the split of Protostomia and Deuterostomia [11], [16]. The early evolution of the metazoan is thought to be associated with the emergence of the nervous system. Thus, the phylogenetically basal position of Ngb in the metazoan tree would suggest that it emerged with the evolution of the nerve system [16]. The aim of this study was to determine if Ngb belongs to the basic toolkit that defines the ancestral nervous system and thus is also widely present in invertebrates. To find potential orthologs of Ngb, phylogenetic and genomic analyses were conducted.

### Phylogenetic Analysis

**Figure 1:**
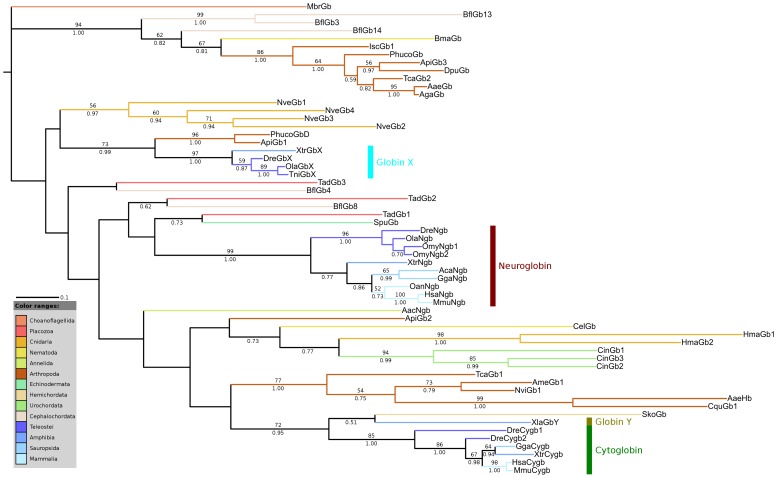
Bayesian tree of Ngb, GbX, Cygb, GbY and several invertebrate globins. Posterior probability values are shown below branches while bootstrap support values were superimposed above the branches. Values above 50% are shown. A globin form the choanoflagellate *Monosiga brevicollis* was used to root the tree. For a description of used abbreviations please see Table S1.

Similarity searches for putative Ngb proteins resulted in the identification of several invertebrate candidate protein sequences. Representative protein sequences of GbX and Cygb were added to the dataset since they clustered with some of the invertebrate candidate proteins. A globin from the choanoflagellate *Monosiga brevicollis* was included to root the tree. The choanoflagellates are the closest relatives of animals and emerged before the split of Protostomia and Deuterostomia. A multiple sequence alignment was created using MUSCLE and refined manually. Various phylogenetic trees were constructed by running neighbor-joining, maximum likelihood algorithms and Bayesian interference. To improve the alignment we excluded the variable N- and C-terminal parts of the proteins, aligning only the globin domains. The alignment is provided in Dataset S1. The final phylogenetic trees derived from maximum likelihood and from Bayesian analyses were nearly identical. Figure 1 shows the Bayesian tree of globin domains from several invertebrate and vertebrate globin proteins with superimposed Bayesian posterior probability and bootstrap support values. The maximum likelihood tree is provided in Figure S1. The topology of the neighbor joining tree slightly differs from these trees, but clustering of the major clades is similar. A figure of the neighbor joining tree is also available in Figure S2. Clustering of the protein sequences in the tree mostly agrees with the species tree. However, discrepancies from the species tree can be found in the clade comprising vertebrate Cygb sequences, e.g. XtrCygb of the amphibian *Xenopus tropicalis* groups together with GgaCygb of the chicken. This is most likely caused by the high sequence similarity of each globin type among vertebrates due to the exclusion of the variable N- and C-terminal parts of the proteins, using only the highly conserved globin domains for tree reconstruction. Interestingly, a globin from the acorn worm *Saccoglossus kowalevskii* (SkoGb) clusters with globin Y from *X. laevis* (XlaGbY) and vertebrate Cygbs. It has been proposed that GbY emerged through a tandem gene duplication before the first round of whole genome duplication in the stem lineage of vertebrates [15]. The close relationship of SkoGb and XlaGbY supports this hypothesis and the ancestry of vertebrate GbY. Recently, we found putative orthologs of GbX in several invertebrate species [12]. Here, the close relationship of GbX with globin proteins from the human body louse and the pea aphid (PhucoGbD, ApiGb1) was recovered, thus supporting our recent findings. This branch received a moderate bootstrap support (73%) and was found in all analyses. Interestingly, three globin proteins from the cephalochordate *B. floridae* (BflGb3, BflGb13, BflGb14) cluster with a globin from the nematode *Brugia malayi* (BmaGb) and several arthropod globins (IscGb1, PhucoGb, ApiGb3, DpuGb, TcaGb2, AaeGb, AgaGb) with high bootstrap support (94%). BflGb3, BflGb13 and BflGb14 represent a distinct class of globin proteins which seem to have originated through a duplication event of an ancestral GbX gene [12], [47]. The putative orthologous relationship of BflGb3, BflGb13 and BflGb14 with several protostome globins supports the hypothesis that the duplication event that gave rise to two distinct copies of GbX predates the bilaterian radiation. As previously shown, the globin proteins from the urochordate *Ciona intestinalis* cluster in a monophyletic group, thus supporting the view that the *Ciona* globins are not 1∶1 orthologous to vertebrate globin genes [47], [48]. Surprisingly, the globin proteins from two cnidarian species, *Nematostella vectensis* and *Hydra magnipapillata*, cluster in clearly separated monophyletic clades. This suggests that different evolutionary forces and species-specific duplications shaped the globin gene repertoire of these two closely related cnidarian species.

**Figure 2:**
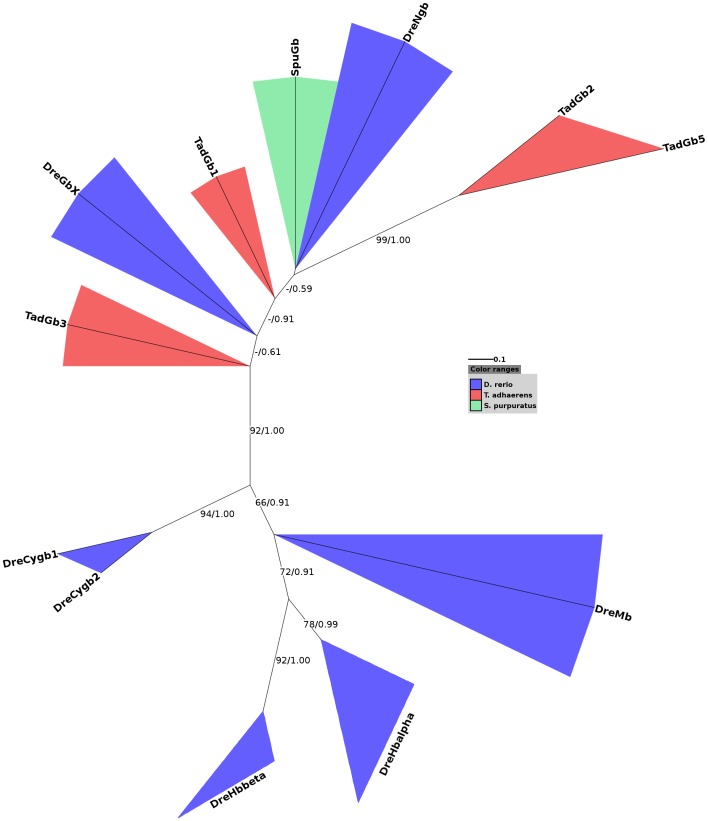
Maximum likelihood tree of several globins from *D. rerio*, *Trichoplax* and the sea urchin. Globins from *D. rerio*, *Trichoplax* and the sea urchin are highlighted in blue, red and green, respectively. Only one globin domain of the polymeric globin from the sea urchin was used for tree reconstruction. Two globins from *Trichoplax* (TadGb4, TadGb5) were excluded from this analysis due to doubtful annotation. The RAxML rapid bootstrapping algorithm was used applying 1000 bootstrap replicates. Bayesian posterior probability values are superimposed (bootstrap support/posterior probability). Only support values above 50% are shown. The clades of α/β-Hb were collapsed. SpuGb and TadGb1, that are located in similar gene neighborhoods as vertebrate Ngb, position next to Ngb and between Ngb and GbX, respectively. For an explanation of abbreviations please see Table S1.

Interestingly, a globin from *Trichoplax* (TadGb1) and the partial polymeric globin protein from the sea urchin (SpuGb) cluster with vertebrate Ngb. We used only one globin domain of the polymeric globin from the sea urchin for tree reconstruction given that it consists of sixteen globin domains and thus is nearly seventeen times longer than human Ngb. However, the branching is neither supported by bootstrap nor by Bayesian posterior probability values, which makes it impossible to draw clear conclusions. The low bootstrap and Bayesian support, especially at the inner branches, is caused by highly diverged sequences. Moreover, we included several protostome globin sequences in our data set that have multiple origins and thus group polyphyletically in trees. Interestingly, after exclusion of all protostome globin proteins from our data set a clustering of the partial SpuGb and Ngb was observed. Figure 2 illustrates the maximum likelihood tree of the partial SpuGb, the globin proteins of *Danio rerio* and the putative globin proteins of *Trichoplax* with superimposed bootstrap and Bayesian posterior probability values. Two putative globins from *Trichoplax* were excluded from our analyses due to ambiguous gene structures. All included globins of *Trichoplax* cluster with Ngb and GbX with high bootstrap support (92%). Interestingly, TadGb1 that previously clustered with SpuGb positions between Ngb and GbX in the narrowed phylogenetic tree. The close relationship of SpuGb and TadGb1 with vertebrate Ngb suggest that those globins may be orthologous and that an Ngb-like protein may be present in invertebrates. Interestingly, a close relationship of SpuGb and vertebrate Ngbs has lately been proposed by Bailly and Vinogradov [49]. Moreover, recently a globin protein (BflGb4) orthologous to Ngb has been identified in the cephalochordate *B. floridae* [GenPept: XP_002589215.1] [47]. This observation is not clearly supported in our main trees (Figure 1) in which BflGb4 clusters with a putative globin from *Trichoplax* (TadGb3) rather than with vertebrate Ngb. However, this branch is neither well supported by bootstrapping nor by Bayesian posterior probability. Interestingly, after exclusion of all protostome globin proteins from the analysis a clustering of BflGb4 with Ngb was observed (tree not shown).

In contrast to its high conservation in the vertebrate lineage, Ngb seems to be only present in a few invertebrate taxa. The slow evolutionary rate of vertebrate Ngb indicates an important function of this protein that it may have gained after the split of Urochordata and Chordata [45].

### Synteny Analysis

**Figure 3:**
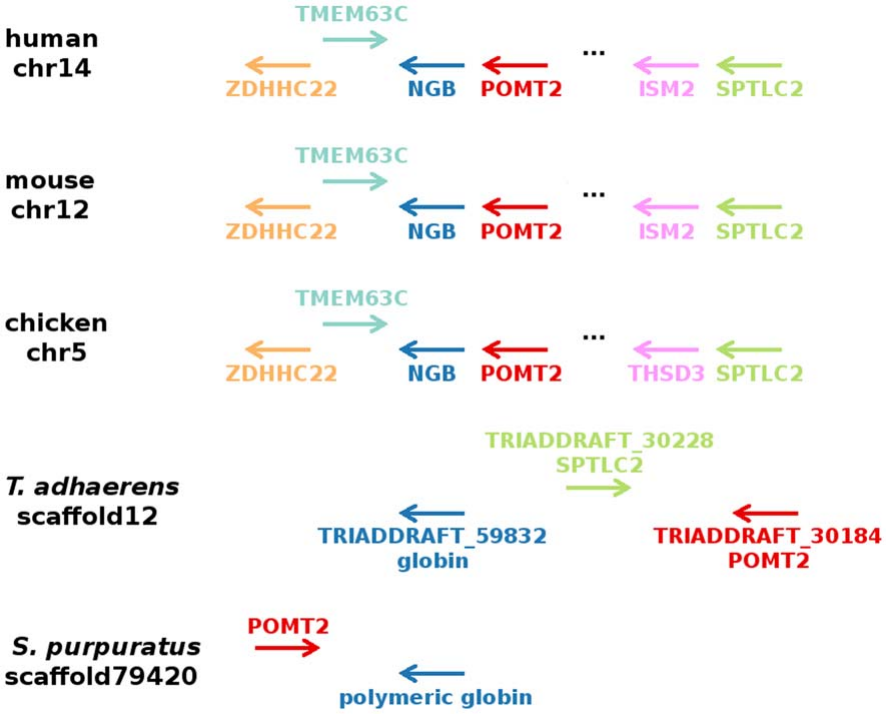
Gene neighborhood of vertebrate Ngb compared to the genomic localization of the putative invertebrate orthologs. Shown is the gene neighborhood of Ngb from human, mouse and chicken compared to scaffold 12 of the *Trichoplax* genome and scaffold 79420 of the sea urchin genome. Arrows indicate the location of the genes (right handed arrow  =  plus strand, left handed arrow  =  minus strand). Genes drawn in the same color are orthologous. Dots indicate that shown genes are separated by more than one gene. The shared syntenic position of the globin of *Trichoplax*, the polymeric globin from the sea urchin and of vertebrate Ngb indicates that they are orthologous.

To find further evidence for the orthology among TadGb1, SpuGb and Ngb and to identify additional orthologs, the gene neighborhood of vertebrate Ngb was analyzed and compared to invertebrate species. During evolution rearrangements of the genome can lead to loss and gain of colinearity between two loci. Shared synteny is one of the most reliable criteria to prove the orthology of genomic regions in different species, as it hints to a possible common origin. The chromosomal position of Ngb is highly conserved in Amniota, showing a preserved co-localization of several genes with Ngb (Figure 3) [50], [51]. The human Ngb gene is located on chromosome 14 between the genes coding for POMT2 and TMEM63C. Srivastava and her colleagues demonstrated that the genome of *Trichoplax* shows large blocks of conserved synteny relative to the human genome [52]. *Trichoplax* is the only named species in the phylum Placozoa and presents the simplest free-living animal. Interestingly, the putative ortholog of POMT2 is adjacent to the TadGb1 gene (TRIADDRAFT_59832) on scaffold 12 (Figure 3). Such a preserved co-localization was also observed in the genome of the sea urchin in which the putative ortholog of POMT2 is adjacent to the polymeric globin SpuGb. The shared synteny strongly suggests a common origin of these particular globin genes and vertebrate Ngb. Unfortunately, we were not able to detect a preserved co-localization in any of the additional analyzed genomes (see material and methods). Nevertheless, sequencing of several analyzed genomes is still going on and assembling scaffolds into chromosomes may lead to the identification of further globin genes sharing a preserved genomic neighborhood with vertebrate Ngb.

The phylogenetic analysis and the shared synteny between the globin of *Trichoplax* (TadGb1), the polymeric globin of the sea urchin (SpuGb) and vertebrate Ngbs provide convincing evidence that these globin genes are orthologous. Interestingly, the orthologous relationship between Ngb and the polymeric globin from the sea urchin has just recently also been described by Hoffmann and colleagues [53]. We further propose that Ngb is present in the placozoan *Trichoplax*. Recently Loenarz et al. reported that the main components of the human hypoxia-inducible transcription factor (HIF) system function in *Trichoplax* as an adaptation to the changing oxygen levels in the early Cambrian period [54]. It has been suggested that vertebrate Ngb protects neurons from hypoxia-related stress [36], [37]. Thus, although *Trichoplax* lacks neuronal cells, the putative orthologous globin protein of *Trichoplax* might function in protecting cells against oxidative stress similarly to Ngb. It will be intriguing to examine if the orthologs of Ngb in the sea urchin and *B. floridae* are already preferentially expressed in neuronal cells or if the neuron-specificity of Ngb emerged with the evolution of the vertebrate nervous system.

### Gene Structure

**Figure 4:**
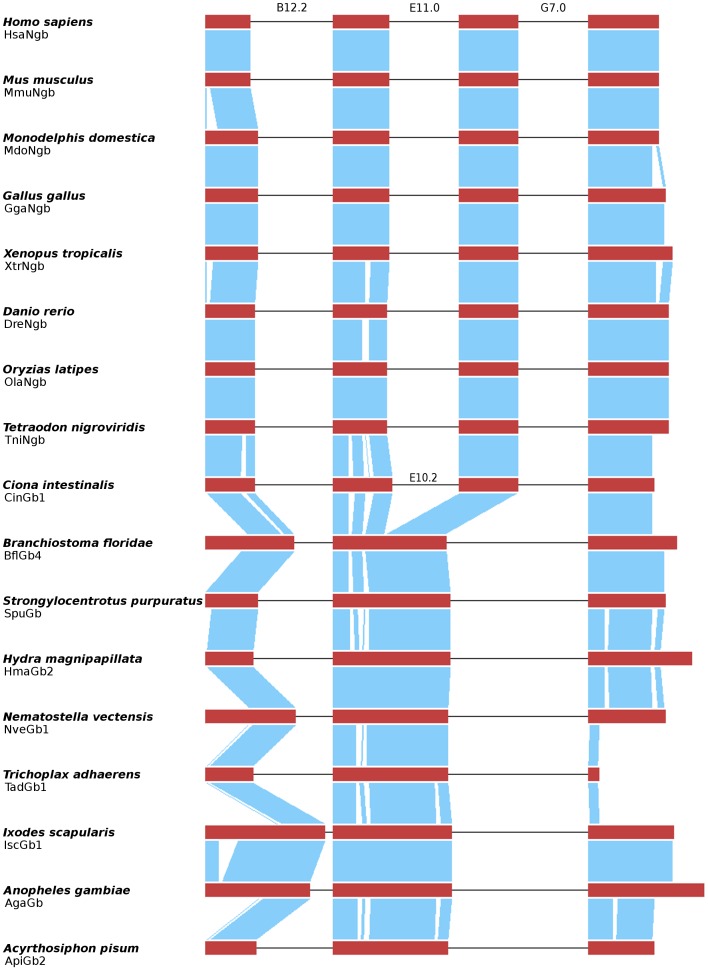
Schematic representation of the coding exon structure of selected vertebrate Ngbs and several invertebrate globins. The coding exons and the corresponding protein alignments are printed in red and blue, respectively. Black lines indicate introns. Vertebrate Ngb genes consist of four highly conserved coding exons and three introns at positions B12.2, E11.0 and G7.0. The globins from *Ciona intestinalis* also possess a central intron at position E10.2. However, this intron is not orthologous to the central intron in Ngb genes. Abbreviations are explained in Table S1.

Most vertebrate and invertebrate globin genes contain two introns at positions B12.2 (i.e. between the second and third base of codon 12 in globin helix B) and G7.0, which are considered as phylogenetically ancient [1], [55]. Vertebrate Ngb genes possess an additional intron at position E11.0 while GbX genes possess two extra introns at positions E10.2 and H10.0 [11], [16]. The presence of introns in the E-helix at slightly different positions in globin genes of vertebrate, invertebrate and plant species raised the question if such a central intron was already present in the globin ancestor or if it was gained several times independently [1], [55], [56]. Moreover, intron positions are useful indicators for the relatedness of globin genes. By comparing the gene structure of globin genes from several invertebrate species, we wanted to determine if the intron at position E11.0 of Ngb was gained in the lineage leading to vertebrates or if this central intron was already present in invertebrates, in particular in globin genes orthologous to Ngb (BflGb4, SpuGb, and TadGb1). Recently, Ebner and colleagues revealed the structure of the globin genes from *B. floridae* and showed that the BflGb4 lacks a central intron [47]. Same applies to the polymeric globin of the sea urchin [49]. We determined the exon/intron structure of putative globin genes from *Trichoplax*, *Hydra magnipapillata*, and the arthropod species that cluster with *B. floridae* paralogs of GbX in our phylogenetic trees. The UTR exons were excluded given that they are not annotated for most genomes used in this study. For clarity, the distributions of the coding sequences (CDSs) of only few representative globin genes are shown in Figure 4. As expected, the CDSs of nearly all analyzed invertebrate globin genes are distributed on three exons, separated by two introns in the phylogenetically ancient positions B12.2 and G7.0 [1], [55]. The globin genes of the two invertebrate chordate species, *Ciona* and *B. floridae*, are exceptions from that. All four Ciona globin genes possess introns at positions B12.2, E10.2 and G7.0, except CinHb2 which has lost the intron at position G7.0 [48]. The amphioxus globin genes have varying structures. Some possess only two introns in ancient positions B12.2 and G7.0 while other contain additional introns at different positions in the E-helix and at positions in the N- and C-terminal part of the protein [47]. Thus, it was proposed that the central introns in globin genes from several taxa were acquired by convergent intron gain. Previously it was shown for the insect lineage that the original globin gene harbored introns at the ancient positions B12.2 and G7.0 and that introns in deviating positions are products of recent intron loss and intron gain events [57]. The hypothesis of two ancestral introns in positions B12.2 and G7.0 is further supported by the absence of a central intron in globin genes from cnidarian, echinoderm [49] and placozoan species. Further, the lack of the E11.0 intron in invertebrate Ngb orthologs confirms an intron gain event in the Ngb gene in the stem lineage of vertebrates.

### Ka/Ks Ratio

**Figure 5:**
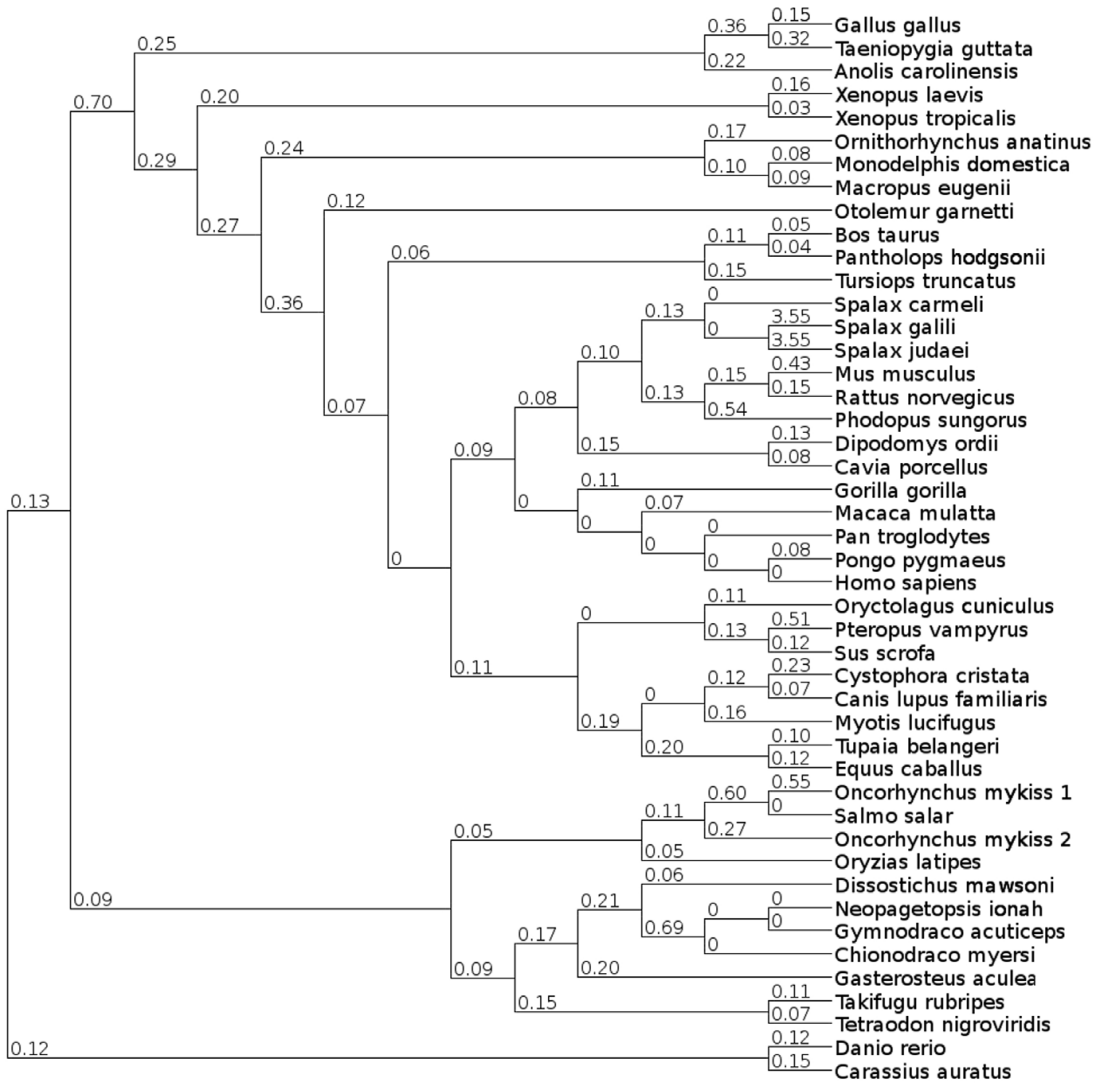
Ka/Ks ratios of several vertebrate Ngb proteins. Shown is a neighbor joining tree with Ka/Ks ratios indicated above the branches. Expect for branches leading to *S. galili* and *S. judaei*, no values above one were observed indicating strong purifying selection.

**Figure 6:**
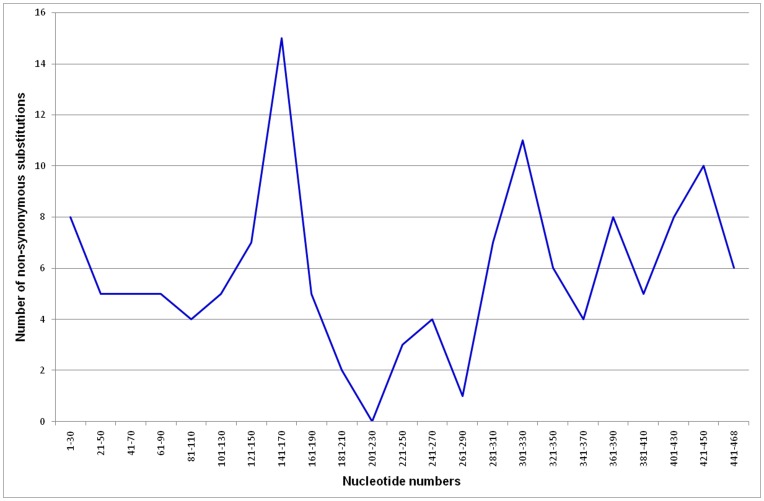
Distribution of missense mutations over the coding sequences of Teleostei fishes. The ancestral sequence of Tetrapoda and Teleostei was used as a reference. The sum of nonsynonymous substitutions was calculated for each 30 nucleotides.

The ratio of the number of nonsynonymous substitutions per nonsynonymous site (Ka) to the number of synonymous substitutions per synonymous site (Ks) is often used as an indicator of selective forces acting on a protein. Usually Ka less than Ks (Ka/Ks <1) suggests purifying selection acting on the protein. In contrast a Ka/Ks ratio greater than one (Ka/Ks >1) gives strong evidence for positive selection causing a change of the protein. A Ka/Ks ratio around one indicates neutral evolution. We used the Fuge Bioinformatics platform (http://services.cbu.uib.no/tools/kaks) to reconstruct the ancestral sequences of vertebrate Ngbs and to calculate Ka/Ks ratios for each branch of the phylogenetic tree (Figure 5). A Ka/Ks ratio much greater than one was only observed for the branch leading to *Spalax judaei* and *S. galili*. However, this is a result of the close relationship of the two species and a Ks value of zero. All other branches show a Ka/Ks ratio from zero to 0.67, indicating strong purifying selection. Among few outliers, the highest value (0.67) was observed on the branch leading to Tetrapoda. This may suggest that positive selection acted on the Ngb protein as the Gnathostomata diverged. We used the ancestral sequence of Tetrapoda and Teleostei as reference to identify regions of evolutionary sequence variation in the Ngb coding sequences of the Teleostei fishes. For this we counted the number of missense mutations in the coding sequences of several Teleostei species compared to the ancestral nucleotide sequence. The graph shows that the CD region is the fastest evolving part of the fish Ngb proteins in contrast to a region between the E and F helix (heme pocket), which is highly conserved (Figure 6). Interestingly, it was shown recently that substitutions in the CD region account for alterations in conformational mobility of the icefish *Chaenocephalus aceratus* Ngb [58]. Thus, the higher evolutionary rate of the CD region of fish Ngbs may point to some functional differences between teleost fish and terrestrial vertebrates as an adaption to their lifestyles.

### Phylogenetic Footprinting

**Figure 7:**
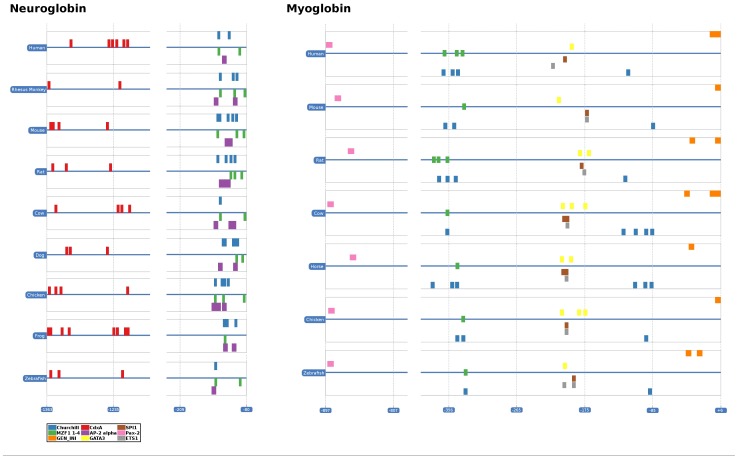
Graphical overview of the results from the CONREAL search. The TFBSs are represented as blocks with different colors. The blue lines indicate the genomic sequence. The scale on the x-axis describes the position of the motifs relative to the translation initiation site. The predicted TFBSs are positionally conserved among the different analyzed vertebrates.

**Figure 8:**
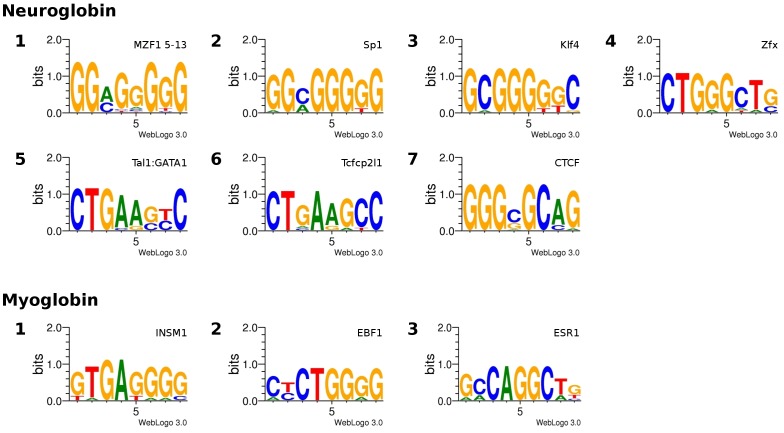
Results of the FootPrinter motif search. Sequence logos of found motifs in Mb and Ngb genes are shown. The found motifs are G-rich and highly conserved. According to the comparison against the JASPAR database the motifs show the highest similarity to binding sites of TFs highlighted above the logos. The results of the complete phylogenetic footprinting analyses are summarized in Table1.

Although the function of Ngb has been widely discussed, only little is known about the modes of gene regulation underlying its cellular function. In order to identify candidate gene regulatory sequences, we applied phylogenetic footprinting to search for the distribution and interspecific conservation of potential transcription factor binding sites (TFBSs). To find TFBSs that control the expression specificity of Ngb, a comparative search for TFBSs in the Mb gene was also performed. Phylogenetic footprinting is a method for the prediction of transcription factor binding sites in a set of orthologous sequences. The method is based on the assumptions that functional regions of genes evolve at a slower rate than the non-functional surrounding sequence and that orthologous genes are controlled by the same regulatory mechanisms in different species. Three different tools were applied (MEME, CONREAL, FootPrinter) with very strict parameters to reduce the number of false positives. The results are summarized in Table 1. We detected several conserved motifs in the upstream sequences of both analyzed globin genes. The CONREAL search revealed the presence of five and seven positionally conserved TFBSs for Ngb and Mb, respectively. Figure 7 shows the distribution of the different motifs among the analyzed species. A table with detailed results is provided in Table S4. FootPrinter detected ten and four motifs (Figure 8, Table S3) of eight bp length in the Ngb and Mb genes, respectively. The reported motifs are G-rich and highly conserved, though not at the same positions. Additionally, MEME was used to detect further conserved motifs. A search for the ten best motifs with a maximal length of fifteen bps using either un-weighted or weighed sequences was done (Figure S3, Table S2a). The detected motifs were not positionally conserved. The statistical significance was verified by repeating the same analysis with shuffled sequence letters (Table S2b). The motifs predicted by FootPrinter and MEME were matched against the JASPAR database to check for similarities with validated TFBSs (Table S2a, Table S3). This comparison provided a clue of which transcription factors may be involved in the regulation of the expression of Ngb and Mb. However, many transcription factors that were predicted to bind to these motifs have either a very variable or a strongly differing consensus DNA binding site. Whether these transcription factors bind to the predicted motifs *in vivo* has to be verified by wet-lab experiments.

**Table 1:** Predicted motifs and possible corresponding transcription factors found via phylogenetic footprinting.

Transcription factor	Ngb	Mb	Function
AP2alpha	+	−	cranial neural crest and limb mesenchyme development [80], [81]
CdxA	+	−	pattering of the anteroposterior axis [117]
Churchill	+	+	mediator of FGF signaling during neural development [68]
CTCF	+	−	insulator activity [118]
EBF1	−	+	B-cell differentiation [70]
ESR1	−	+	development and maintenance of normal sexual and reproductive function [119]
c-ETS	−	+	embryonic and hematopoietic development; angiogenesis [83]
GATA-3	−	+	expressed in T cells in adult animals; expression in non-hematopoietic tissues/organs during embryogenesis [120]
Inr	−	+	RNA polymerase II transcription initiation [121]
INSM1	+	+	neuroendocrine differentiation [69]
IRF1/IRF2	+	−	development and activation of immune cells; IRF2 suppresses transcriptional activity of IRF-1 [122]
Klf4	+	−	development; differentiation; maintenance of tissue homeostasis [123]
MZF1 1–4 (domain 1)	+	+	hemopoietic development; differentiation of myeloid cells [72], [73], [74]
MZF1 5–13 (domain 2)	+	−	
NR3C1	−	+	inflammatory response; cellular proliferation; differentiation in target tissues through interaction with steroidal hormones (Glucocorticoids) [124]
Pax2	−	+	kidney development; establishing mid-hindbrain border [71]
Pax4	+	+	organogenesis of the pancreas [71]
Pax5	+	+	B-cell differentiation; neural development [71]
PPARG:: RXRA	−	+	adipocyte differentiation; lipogenesis [125]
REST	+	−	repression of neuronal genes in stem cells and non-neuronal tissues [126]
RREB1	−	+	ubiquitously expressed repressor [79], [127]
RXR::RAR_DR5	+	−	embryonic development; tissue homeostasis; cell proliferation; differentiation; apoptosis [128]
Sox2	−	+	progression of neurogenesis; maintenance of neurons [129]
Sp1	+	−	trans-activator; ubiquitously expressed [61]
SPI-1 (PU.1)	−	+	differentiation of myeloid cells, B-cells, macrophages [130]
Tal1:GATA-1	+	−	erythroid differentiation [82]
Tcfcp2l1	+	−	transcriptional repressor [131], [132]
Zfx	+	−	self-renewal and maintenance of embryonic and hematopoietic stem cells [133]
znf143	−	+	trans-activation of small nuclear RNA (snRNA) and snRNA-type promoters [134]

In agreement with previous studies [16], [51], a putative Sp1 (specific protein 1) binding site was detected in the Ngb gene. Two studies verified recently that Sp1 binds to this site and transactivates the Ngb expression in humans and mice [59], [60]. The trans-activator Sp1 is ubiquitously expressed, binds with high affinity to GC-rich motifs and can activate or repress transcription in response to physiological and pathological stimuli [61]. Another transcription factor for which putative binding sites in the Ngb gene have been previously reported is the neuron-restrictive silencer factor (NRSF), also known as REST [51], [62]. NRSF mediates the neuron-specific expression of several genes by binding to a 21 bp consensus element (NRSE) [63], [64]. MEME reports a relaxed 15 bp motif, located −661 to −647 upstream of the translation start codon ATG of human Ngb, that shares some similarity with experimentally validated NRSF binding sides (motif 10 (u) in Figure S3, Table S2a). We applied further analyses, described in the methods section, to search for the full-length NRSE in the Ngb gene. In contrary to previous studies done by Laufs et al. and Wystub et al. [51], [62], we were not able to detect a full-length NRSE. Interestingly, in the ENCODE project a NRSE site was found in the first intron of human Ngb by a ChIP-seq analysis of pancreatic carcinoma cells [65]. However, the score is quite low and a total of twelve different factors were found to bind at this position. Since most of this region overlaps with a simple repeat these binding sites may be false positives resulting from non-specific protein binding in the ChIP-seq experiments. Interestingly, the other part of the binding sites comes from the exapted MIR element in agreement with the ScrapYard hypothesis [66]. Nevertheless, associated histone modifications (H3K4me1, H3K4me3) that were determined in eight different human non-neuronal cell lines indicate that this region may in fact have enhancer activity [65]. According to this study no other regions of the human Ngb gene are marked by these histone modifications including the relaxed NRSE found by MEME. Recently, Zhang et al. analyzed two putative NRSEs that lie in the promoter region of human Ngb. One of these sites overlaps with the relaxed 15 bp motif found by MEME. The mutation of both NRSEs didn’t lead to an increase in promoter activity, neither in HeLa nor in SH-SY5Y cells [59]. However, these results are not conclusive and it is not clear at this point if NRSE inhibits the expression of Ngb in non-neuronal cells and whether it binds to the shortened motif found by MEME.

Plaisance et al. reported that REST inhibits the Sp1-mediated activation and that Sp1 is required for the expression of REST target genes in cells where REST is absent [67]. So, it is tempting to suggest that the interplay of REST and Sp1 leads to the neuron specific expression of Ngb. Accordingly in non-neuronal cells REST would then interact with Sp1 and prevent the transcriptional activation of Ngb in contrast to neuronal cells where REST is absent.

A comparison of Ngb and Mb revealed five transcription factors that potentially bind to both genes (Churchill, INSM1, Pax4, Pax5 and MZF1 1–4). Churchill is a transcriptional activator that mediates FGF signaling during neural development [68], while INSM1 is an important regulator during neuroendocrine differentiation [69], [70]. The Pax transcription factors play important roles in early development and have been implicated as regulators of organogenesis and as key factors in maintaining pluripotency of stem cell populations [71]. However, the latter are most likely false positives, due to highly variable consensus-DNA binding sites and their function in development and stem cell maintenance.

The myeloid zinc finger protein (MZF1) contains thirteen C2H2 zinc fingers arranged in two domains (domain 1: zinc finger 1–4, domain 2: zinc finger 5–13) and regulates the hemopoietic development and the differentiation of myeloid cells [72], [73], [74]. It has been shown that MZF1 binds to the 5′ boundary area of the human β-globin locus control region (LCR) [75]. Thus, it is plausible that MZF1 is also involved in the regulation of other globin genes like Mb or Ngb. Besides MZF1, further predicted transcription factors are known to bind to other globin genes. AP2α, SP1 and c-ETS likely regulate the expression of Cygb [51], [76]. SP1 is also involved in the transcriptional activation of the Mb and ε-globin genes [77], [78]. GATA1 is an important regulator of the β- and α- globin gene clusters [77], while the transcriptional repressor RREB1 inhibits the ζ-globin promoter [79]. These transcription factors are candidate regulators of the expression of other globin genes like Ngb or Mb. We found potential binding sites for AP2α and GATA1 in the upstream sequence of the Ngb gene and potential binding sites for c-ETS and RREB1 in the upstream sequences of Mb. AP2α is involved in cranial neural crest and limb mesenchyme development in mice [80], [81] while GATA1 acts together with Tal1 as positive regulators of erythroid differentiation [82]. The transcription factor ETS1 (c-ETS) is involved in embryonic and hematopoietic development as well as in angiogenesis [83]. Aside from that we detected additional binding sites for the transcription factors CdxA, Klf4, Zfx, IRF1/IRF2, the transcriptional repressor Tcfcp2l (also referred to as CRTR-1, LBP-9) and the complex RXR::RAR_DR5 that may be unique to Ngb compared to other globin genes.

To sum up, our analysis suggests a complex regulation of the Ngb gene. We have found potential binding sites for MZF1, AP2α, SP1 and GATA-1 that are already known to regulate other globin genes. It is plausible that those transcription factors are also involved in the regulation of Ngb genes. Other predicted factors, e.g. CdxA, Klf4 and Zfx and REST, probably regulate no other globins but Ngb. Thus, they are candidates for dictating the specific expression of Ngb. While REST is a likely candidate that may be responsible for the specific expression of Ngb in the central and peripheral nervous system, emerging evidence suggests that more complex, neuron type specific transcriptional regulation governs neuroglobin expression [26], [27], [28], [30]. Our results directly suggest wet-lab experiments, which could confirm or refute predicted TFBSs.

### Conclusions

Our results show that TadGb1, a putative globin of the placozoan *Trichoplax adhaerens*, as well as the polymeric globin from the sea urchin, are orthologous to vertebrate Ngb. Interestingly, the presence of an Ngb-like gene in a simple animal that lacks neuronal cells suggests that Ngb gained its neuron-specificity later in its evolution, likely via neofunctionalization of duplicated globin genes. In all phylogenetic trees the vertebrate globin proteins were separated by several invertebrate globin proteins supporting the hypothesis that at least three different globin types were present in the last bilaterian common ancestor. Importantly, several putative conserved transcription factor-binding sites were found that likely contribute to the specific expression of Ngb, e.g. CdxA, Klf4 and Tcfcp2l1 and REST. However, wet-lab experiments are required to confirm biological activity of predicted binding sites. Subsequent dissection of gene regulation modes that are specific to Ngb is likely to help decipher its cellular function.

## Materials and Methods

### Sequence Data

The BLASTp algorithm [84] was employed to search the non-redundant protein database of NCBI for homologous globin proteins of invertebrates, using human and zebrafish Ngb as query sequences [GenPept: NP_067080.1, NP_571928.1]. The parameters used were E-value threshold: e-05; word size: three; amino acid substitution matrix: BLOSUM45; gap opening cost: eleven; gap extension cost: one. The received sequences were used as a query for a reverse BLASTp search with the same parameters. A table of sequences used in the consecutive analyses is provided in Table S1.

### Globin Domain Prediction

The normal mode of SMART, including the Pfam database, was employed to predict the globin domains of the analyzed sequences [85] (http://smart.embl-heidelberg.de/).

### Multiple Sequence Alignment

The globin domains were aligned by using the MUSCLE program [86] (http://www.ebi.ac.uk/Tools/muscle/index.html). Subsequently, the alignment was refined manually using Jalview [87].

### Phylogenetic Analysis

The program packages PHYLIP 3.67 [88], RAxML 7.0.4 [89], [90], TREE-PUZZLE 5.2 [91] and MrBayes 3.1.2 [92], [93] were used for phylogenetic tree reconstructions. Phylogenetic analyses were based on the WAG [94] model of amino acid evolution assuming gamma distribution of substitution rates, as suggested by analysis of the alignment with ProtTest3 [95]. Distance matrices were calculated with TREE-PUZZLE. Neighbor-joining trees were inferred using NEIGHBOR. The reliability of the branching pattern was tested by bootstrap analysis with 1000 replications, employing SEQBOOT and CONSENSE. Maximum likelihood analyses were performed using the rapid bootstrapping RAxML algorithm with 1000 bootstrap replications. The Bayesian interference was conducted using MrBayes 3.1.2. Metropolis- coupled Markov chain Monte Carlo sampling was performed with one cold and three heated chains that were run for 10,000,000 generations. The trees were sampled every 1000^th^ generation and the ‘burn in’ was set to 25%. For the calculation of the Bayesian trees, the CIPRES project server was used (CIPRES Web Portal V1.15) [96] (http://www.phylo.org/portal/). The phylogenetic trees were visualized with iTOL [97].

### Synteny Analysis

To assess the synteny conservation among chromosomal segments that harbor Ngb, neighboring genes of Ngb from Homo sapiens, Macaca mulatta, Mus musculus, Bos taurus, Sus scrofa, Canis lupus familiaris, Ornithorhynchus anatinus, Monodelphis domestica, Gallus gallus, Xenopus (Silurana) tropicalis and Danio rerio were identified using the UCSC genome browser and the NCBI Map Viewer [98], [99]. Additional un-annotated genes were determined using the tBLASTn algorithm [84]. Since shared synteny is one of the most reliable criteria to prove orthology of genomic regions, we analyzed the genomic neighborhood of several invertebrate globin genes from Amphimedon queenslandica (Porifera), Trichoplax adhaerens (Placozoa), Nematostella vectensis, Hydra magnipapillata, Acropora digitifera (Cnidaria), Aplysia californica (Mollusca), Schistosoma mansoni, S. japonicum, Schmidtea mediterranea (Platyhelminthes), Caenorhabditis elegans (Nematoda), Strongylocentrotus purpuratus (Echinodermata), Saccoglossus kowalevskii (Hemichordata), Ciona intestinalis and Oikopleura dioica (Urochordata). To identify additional un-annotated invertebrate globin genes, we conducted a tBLASTn search using Ngb, Pomt2 (protein-O-mannosyltransferase 2) or Tmem63C (transmembrane protein 63C), which are located directly down- and upstream of vertebrate Ngb, from D. rerio as query sequences. Orthologous relationships between genes of interest were recognized as reciprocal best hits (RBH) using the BLAST algorithm.

### Exon/Intron Structure Determination

To determine the exon structure and the copy number of invertebrate globin genes, a tBLASTn search was done. The parameters used were E value threshold: 0.001; soft masking of query sequences; no composition-based statistics. Splign [100] and BLAT [101] were used to refine the exon structure. The exon structure was mapped onto the protein alignment using an in-house tool (http://www.bioinformatics.uni-muenster.de/tools/alignstrdraw/).

### Calculation of Ka/Ks Ratio

A phylogenetic tree of 46 vertebrate Ngb proteins comprising all major groups (teleost fishes, amphibians, reptiles, birds, mammals) was created using MUSCLE and the program package PHYLIP 3.67 (for used parameters, see section Phylogenetic Analysis). The Fuge Bioinformatics platform was used with default parameters to calculate Ka and Ks values for each branch (http://services.cbu.uib.no/tools/kaks). The Ka/Ks ratios were manually drawn on the phylogenetic tree. To infer ancestral nucleotide sequences, Ancestor 1.0 with default parameters was applied [102]. To identify regions of evolutionary sequence variation, we counted the number of missense mutations compared to the ancestral nucleotide sequence using a sliding window approach with a window size of thirty nucleotides and steps of twenty nucleotides.

### Phylogenetic Footprinting

Several tools were applied for the identification of conserved putative transcription factor binding sites (TFBSs) among different species. To find TFBSs that control the specific expression of Ngb, a comparative search for TFBSs in the Mb gene was conducted. The Mb gene was chosen because on the one hand it is expressed in different tissues than Ngb and on the other hands it is one of the best functionally studied globins [103], [104], [105]. Genomic sequences comprising 1000 bp upstream of the transcription start site (TSS) of the Ngb and Mb gene plus the 5′UTR and twenty bp of the first coding exon were scanned. This resulted in the analysis of a region comprising 1375 bp and 1172 bp upstream of the translation initiation site (TIS) of Ngb and Mb, respectively. This region is supposed to contain the core and proximal promoter region. The upstream sequences of Ngb of *H. sapiens*, *M. mulatta*, *M. musculus*, *Rattus norvegicus*, *C. lupus familiaris*, *B.* t*aurus*, *G. gallus*, *X. tropicalis* and *D. rerio* and the upstream sequences of Mb of *H. sapiens*, *M. musculus*, *R. norvegicus*, *Equus caballus*, *B. taurus*, *G. gallus* and *D. rerio* were extracted from the UCSC Genome Browser [98]. The MEME program was used to search for conserved motifs with the following parameters: mode oops, number of motifs five, minimum length six and maximal length fifteen [106]. Two different runs were done, one with un-weighted and the other one with weighted sequences. When weights were used, sequences contributed to motifs in proportion to their weights. The weights were set depending on the relationship of the different species, i.e. distant related species (high weights) contributed more to motifs than close related species (low weights). Subsequently, the same analysis was conducted using the option “shuffle sequence letters” to verify the statistical significance of found motifs. In the FootPrinter 3.0 analysis [107] the motif size was set to eight, the maximal number of mutations to three, the maximal number of mutations per branch to one, the subregion change cost to zero, and the subregion size to one-hundred. No alignment guide was used and no regulatory element losses were allowed. WebLogo 3 was used for the visualization of the reported motifs [108] (http://weblogo.threeplusone.com/) and a search against the JASPAR database was conducted to identify vertebrate transcription factors that may bind to these motifs [109]. Furthermore, the CONREAL program was used to search for putative TFBSs that are positionally conserved [110]. The parameters used were: 75–85% threshold for position weight matrices; length of flanks to calculate identity 5 bp; threshold for identity 50–75%. A custom-made perl script was used to compare all pairwise analyses and to extract those TFBSs that are positionally conserved among all species in a range of fifty bp. Finally, RepeatMasker was used to determine if the detected motifs originated from transposable element [111].

### Search for Putative Neuron-restrictive Silencer Elements (NRSEs)

We conducted a pattern search for NRSEs near the Ngb gene of several organisms, comprising a region from 5 kb upstream to 5 kb downstream of the Ngb gene, using a custom-made perl script. The sequences of *H. sapiens*, *M. mulatta*, *M. musculus*, *R. norvegicus*, *C. lupus familiaris*, *B. taurus*, *G. gallus*, *X. tropicalis* and *D. rerio* were extracted from the UCSC Genome Browser [98]. We searched for different consensus sequences of the NRSE that were described in previous studies [112], [113], [114] and for the pattern provided by TRANSFAC (M00256) [115] allowing no mismatches. Additionally, MATCH™ was used to search with different position weight matrixes (V$NRSE_B, V$NRSF_01, V$NRSF_Q4, V$REST_01) for putative NRSEs [116]. The cut-offs were set to minFP. This analysis was repeated for the muscle-specific Mb gene of *H. sapiens*, *M. musculus*, *R. norvegicus*, *Equus caballus*, *B. taurus*, *G. gallus* and *D. rerio* to verify the reliability of the results. Moreover, Chip-seq data from the ENCODE project was analyzed via the UCSC Genome Browser [65].

## Supporting Information

Dataset S1 Alignment of globin domains in interleaved phylip format.
(PHY)

Figure S1 Maximum likelihood tree of Ngb, GbX, Cygb and several invertebrate globins. The RAxML rapid bootstrapping algorithm was used applying 1000 bootstrap replicates. Bootstrap support values above 50% are superimposed. A globin form the choanoflagellate *Monosiga brevicollis* was used to root the tree. For a description of used abbreviations please see Table S1.
(TIF)

Figure S2 Neighbor joining tree of Ngb, GbX, Cygb and several invertebrate globins. The PHYLIP package and TREE-PUZZLE were used applying 1000 bootstrap replicates. Bootstrap values above 50% are shown. A globin form the choanoflagellate *Monosiga brevicollis* was used to root the tree. For a description of used abbreviations please see Table S1.
(TIF)

Figure S3 Results of the MEME motif search. Sequence logos of the found motifs for Ngb and Mb are shown. Motifs 1–10 were found using un-weighted sequences (u) and motifs 11–16 by using weighted sequences (w). Only six out of ten motifs of the analysis with weighted sequences are shown, since the additional ones correspond to motifs 2, 3, 4, 5 and motifs 1, 3, 6, 10 of Ngb and Mb, respectively. The position weight matrixes were used to search for similar TFBSs in the JASPAR database. The TFBSs with highest similarity are highlighted above each logo. The results of the complete phylogenetic footprinting analyses are summarized in Table 1.
(TIF)

Table S1 Table of sequences used in this study.
(DOC)

Table S2 Comprehensive results of the MEME analysis. E-values of the found motifs, p-values, start and end positions (relative to the translation start codon ATG) of the predicted sites are given, as well as the results of the Jaspar search. Additionally, the results obtained by using the “shuffle sequence letter” option are listed.
(DOC)

Table S3 Comprehensive results of the FootPrinter analysis. For each motif the parsimony score, the start and end positions of the sites relative to the translation start codon ATG and the results from the comparison against the Jaspar database are given.
(DOC)

Table S4 Comprehensive results of the CONREAL analysis. The score, relative score, strand, start and end position relative to the translation start codon ATG for each binding site are given.
(DOC)
