# Supplementary figures and images for: Comparative Genomics of Neuroglobin Reveals Its Early Origins
Source: PLoS One. 2012 Oct 25;7(10):e47972. doi: 10.1371/journal.pone.0047972 (PMC3485006; doi:10.1371/journal.pone.0047972)

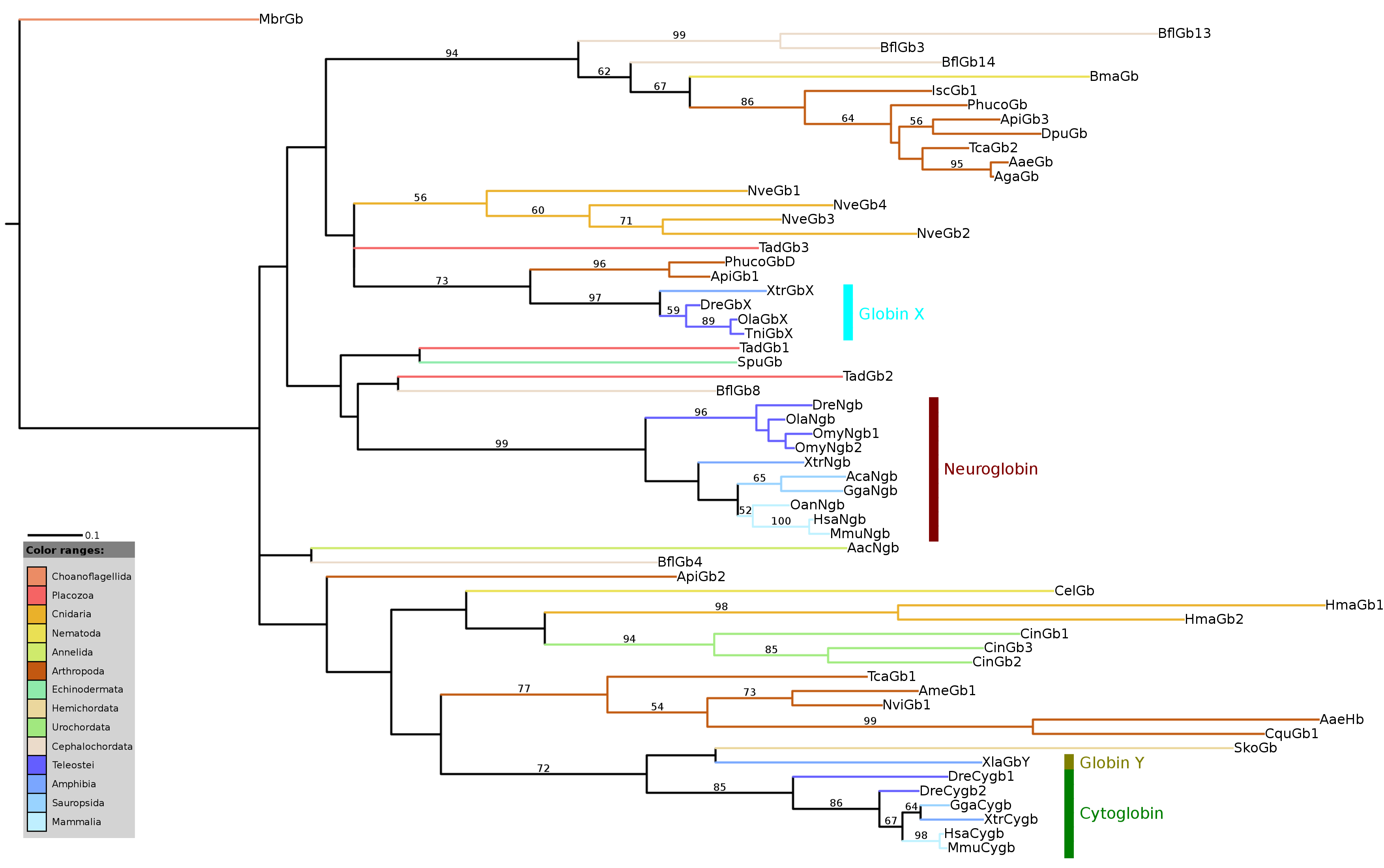

Supplement: Figure S1 — Maximum likelihood tree of Ngb, GbX, Cygb and several invertebrate globins. The RAxML rapid bootstrapping algorithm was used applying 1000 bootstrap replicates. Bootstrap support values above 50% are superimposed. A globin form the choanoflagellate Monosiga brevicollis was used to root the tree. For a description of used abbreviations please see Table S1. (TIF) [file pone.0047972.s002.tif]

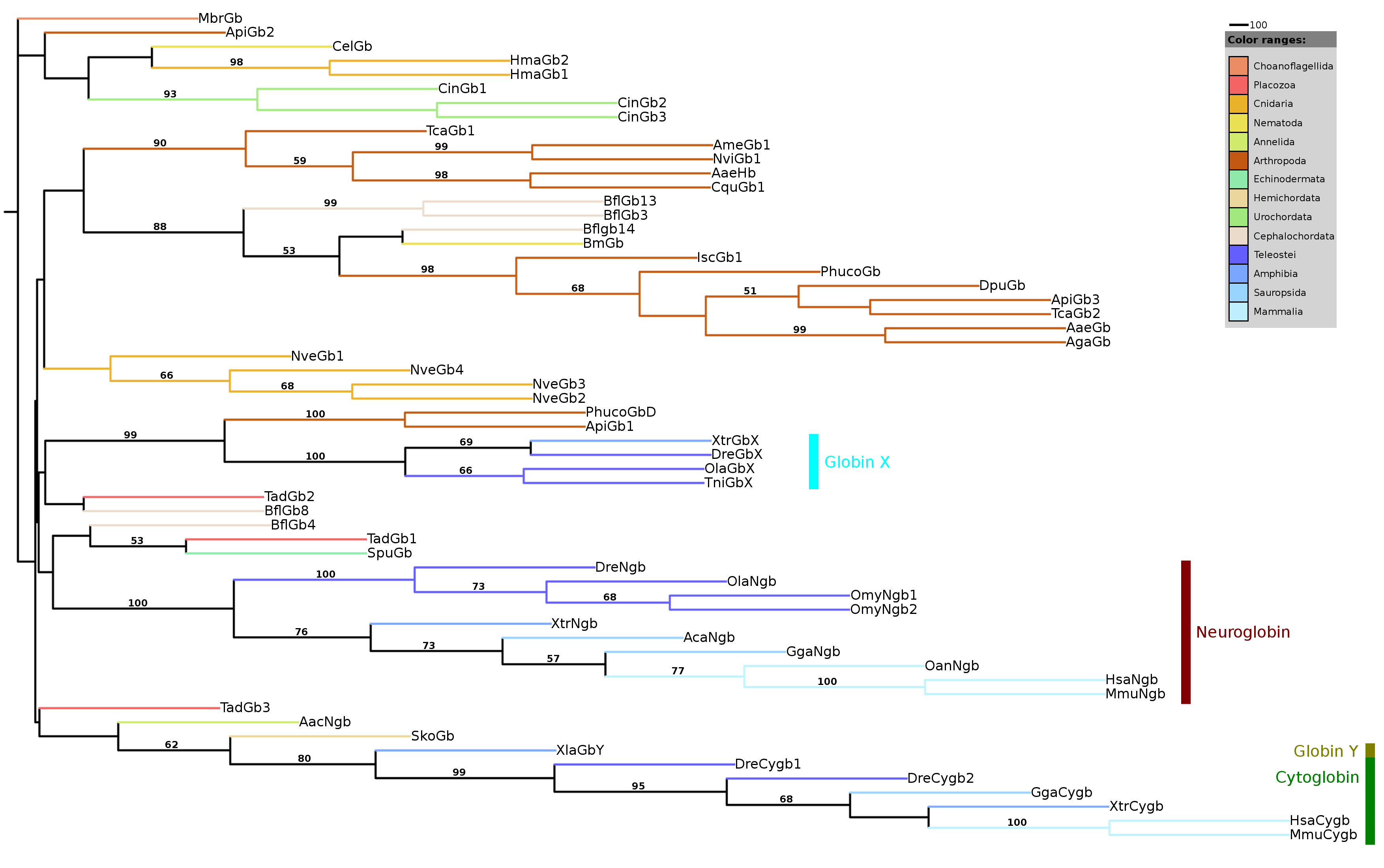

Supplement: Figure S2 — Neighbor joining tree of Ngb, GbX, Cygb and several invertebrate globins. The PHYLIP package and TREE-PUZZLE were used applying 1000 bootstrap replicates. Bootstrap values above 50% are shown. A globin form the choanoflagellate Monosiga brevicollis was used to root the tree. For a description of used abbreviations please see Table S1. (TIF) [file pone.0047972.s003.tif]

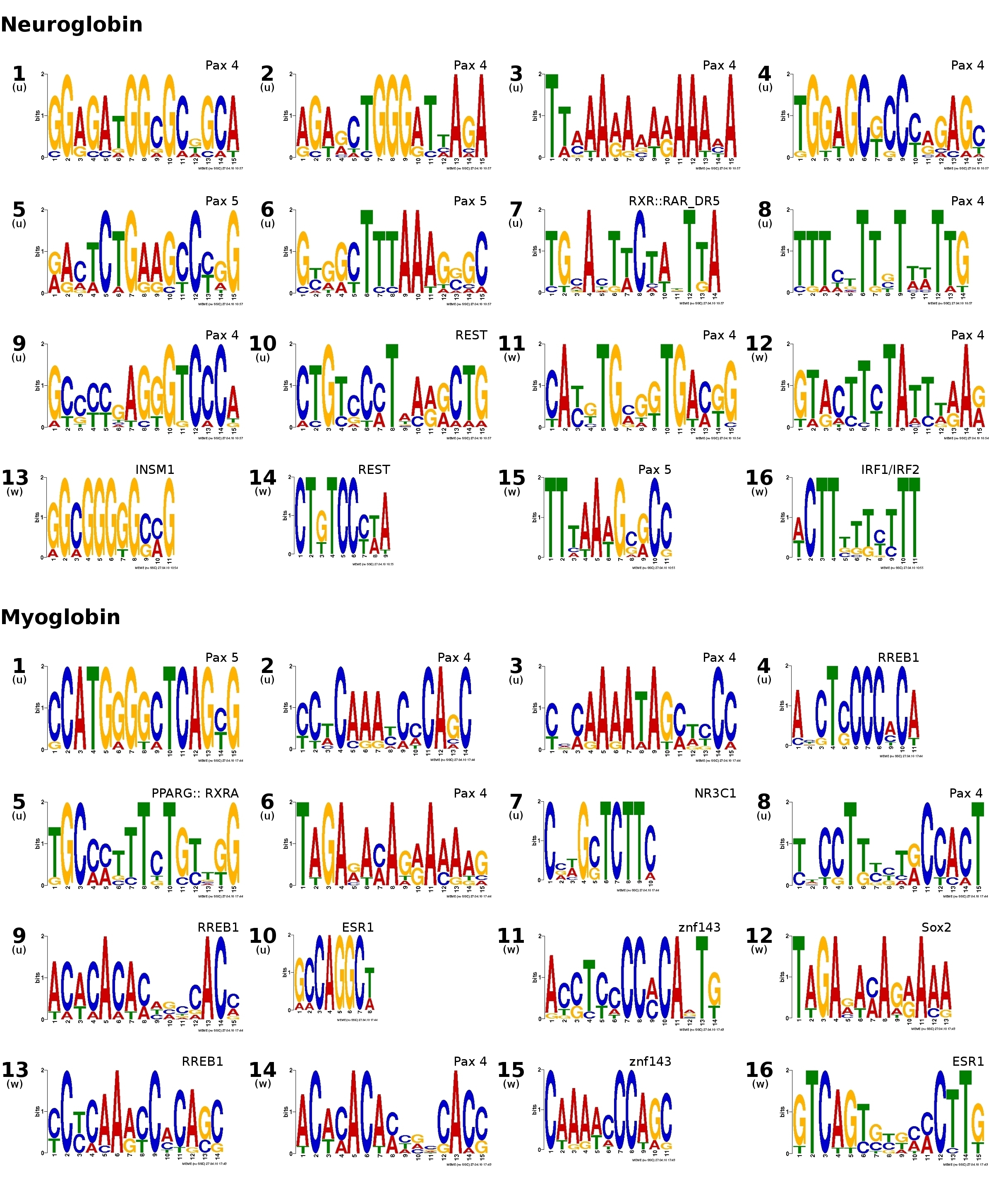

Supplement: Figure S3 — Results of the MEME motif search. Sequence logos of the found motifs for Ngb and Mb are shown. Motifs 1–10 were found using un-weighted sequences (u) and motifs 11–16 by using weighted sequences (w). Only six out of ten motifs of the analysis with weighted sequences are shown, since the additional ones correspond to motifs 2, 3, 4, 5 and motifs 1, 3, 6, 10 of Ngb and Mb, respectively. The position weight matrixes were used to search for similar TFBSs in the JASPAR database. The TFBSs with highest similarity are highlighted above each logo. The results of the complete phylogenetic footprinting analyses are summarized in Table 1. (TIF) [file pone.0047972.s004.tif]
