# Supplementary material for: Comparative Genomics of Neuroglobin Reveals Its Early Origins
Source: PLoS One. 2012 Oct 25;7(10):e47972. doi: 10.1371/journal.pone.0047972 (PMC3485006; doi:10.1371/journal.pone.0047972)
Supplement: Table S1 — Table of sequences used in this study. (DOC) [file pone.0047972.s005.doc]

Table S1: List of used sequences. Sequences that were manual corrected are indicated by *.

| Organism | Common name | globin | Abbreviation | Accession no. |
| --- | --- | --- | --- | --- |
| *Acyrthosiphon pisum* | pea aphid |  | ApiGb1  ApiGb2  ApiGb3 | NP_001191927.1*  NP_001156237.1  XP_001946608.1 |
| *Aedes aegypti* | yellow-fever mosquito |  | AaeHb  AaeGb | XP_001654443.1  XP_001648433.1* |
| *Anolis carolinensis* | green anole | Ngb | AcaNgb | ENSACAP00000016888 |
| *Anopheles gambiae* | African malaria mosquito |  | AgaGb | XP_551936.2* |
| *Aphrodita aculeata* |  |  | AacNgb | AAC47259.1 |
| *Apis mellifera* | honey bee |  | AmeGb1 | NP_001071291.1 |
| *Branchiostoma floridae* | Florida lancelet |  | BflGb3  BflGb4  BflGb8  BflGb13  BflGb14 | XP_002610016.1  XP_002589215.1  XP_002608525.1  XP_002598546.1  XP_002610160.1 |
| *Brugia malayi* | filarial nematode worm |  | BmaGb | XP_001894637.1* |
| *Caenorhabditis elegans* |  |  | CelGb | NP_510079.1 |
| *Ciona intestinalis* | transparent sea squirt |  | CinGb1  CinGb2  CinGb3  CinGb4 | NP_001027700.1  NP_001027699.1  NP_001027698.1  NP_001027701.1 |
| *Culex quinquefasciatus* | southern house mosquito |  | CquGb1 | XP_001870465.1 |
| *Danio rerio* | zebra fish | Ngb Cygb-1  Cygb-2  Mb  GbX  Hb-β  Hb-β  Hb-β  Hb-β  Hb-β  Hb-β  Hb-α  Hb-α  Hb-α  Hb-α | DreNgb  DreCygb1  DreCygb2  DreMb  DreGbX  DreHbba1  DreHbba2  DreHbbe1  DreHbbe1.1  DreHbbe2  DreHbbe3  DreHbaa1  DreHbae1l  DreHbae1  DreHbae3  DreBa2l  Dre445037  Dre563335  Dre497166 | NP_571928.1  NP_694484.1  NP_001019395.1  NP_956880.1  NP_001012261.2  NP_571095.1  NP_001005403.1  NP_001091054.1  NP_571834.1  NP_998011.1  NP_001015058.1  NP_571332.2  XP_685362.2  NP_891985.1  NP_898889.1  CAE30439.1  NP_001003431.1  NP_001076303.1  NP_001013479.1 |
| *Daphnia pulex* | water flea |  | DpuGb | EFX88598.1* |
| *Gallus gallus* | chicken | Ngb Cygb | GgaNgb  GgaCygb | NP_001026722.1  NP_001008789.1 |
| *Homo sapiens* | human | Ngb  Cygb | HsaNgb  HsaCygb | NP_067080.1  NP_599030.1 |
| *Hydra magnipapillata* | hydra |  | HmaGb1  HmaGb2 | XP_002161790.1  XP_002162062.1 |
| *Ixodes scapularis* | black-legged tick |  | IscGb1 | XP_002405154.1 |
| *Monodelphis domestica* | gray short-tailed opossum | Ngb | MdoNgb | NP_001030592.1 |
| *Monosiga brevicollis MX1* |  |  | MbrGb | XP_001744813.1 |
| *Mus musculus* | house mouse | Ngb Cygb | MmuNgb  MmuCygb | NP_071859.1  NP_084482.1 |
| *Nasonia vitripennis* | parasitic wasp |  | NviGb1 | XP_001608300.1 |
| *Nematostella vectensis* | starlet sea anemone |  | NveGb1  NveGb2  NveGb3  NveGb4  NveGb5  NveGb6  NveGb7  NveGb8  NveGb9 | XP_001641645.1  XP_001635310.1  XP_001629477.1  XP_001633127.1  XP_001640985.1  XP_001640562.1  XP_001636079.1  XP_001636078.1  XP_001635635.1 |
| *Oncorhynchus mykiss* | rainbow trout | Ngb-1  Ngb-2 | OmyNgb1  OmyNgb2 | NP_001117860.1  NP_001117861.1 |
| *Ornithorhynchus anatinus* | duck-billed platypus | Ngb | OanNgb | XP_001508417.1 |
| *Oryzias latipes* | medaka fish | Ngb GbX | OlaNgb  OlaGbX | ENSORLP00000020358  ENSORLP00000021338 |
| *Pediculus humanus corporis* | human body louse |  | PhucoGbD  PhucoGb | XP_002427507.1  XP_002426939.1* |
| *Saccoglossus kowalevskii* | acorn worm |  | SkoGb | NP_001161601.1 |
| *Strongylocentrotus purpuratus* | purple sea urchin |  | SpuGb | XP_001199205.1 |
| *Tetraodon nigroviridis* | green puffer | Ngb  GbX | TniNgb  TniGbX | CAC59974.1  CAG25725.1 |
| *Tribolium castaneum* | red flour beetle |  | TcaGb1  TcaGb2 | XP_974723.1  XP_974747.1* |
| *Trichoplax adhaerens* |  |  | TadGb1  TadGb2  TadGb3  TadGb4  TadGb5  TadGb6 | XP_002115923.1  XP_002118316.1  XP_002113619.1  XP_002110659.1  XP_002110660.1*  XP_002113774.1 |
| *Xenopus laevis* | African clawed frog | globin Y | XlaGbY | NP_001089155.1 |
| *Xenopus tropicalis* | Western clawed frog | Ngb Cygb  GbX | XtrNgb  XtrCygb  XtrGbX | NP_001025522.1  NP_001006870.1  NP_001011196.1 |
