# Supplementary material for: Comparative Genomics of Neuroglobin Reveals Its Early Origins
Source: PLoS One. 2012 Oct 25;7(10):e47972. doi: 10.1371/journal.pone.0047972 (PMC3485006; doi:10.1371/journal.pone.0047972)
Supplement: Table S2 — Comprehensive results of the MEME analysis. E-values of the found motifs, p-values, start and end positions (relative to the translation start codon ATG) of the predicted sites are given, as well as the results of the Jaspar search. Additionally, the results obtained by using the “shuffle sequence letter” option are listed. (DOC) [file pone.0047972.s006.doc]

Table S2a: Comprehensive results of the MEME analysis.

|  | **Motif** | **position^1^** | **e-value** | **p-value^2,3^** | | | | | | | | | **Jaspar** | | |
| --- | --- | --- | --- | --- | --- | --- | --- | --- | --- | --- | --- | --- | --- | --- | --- |
|  |  |  |  | **rat** | **mouse** | **rhesus** | **human** | **cow** | **dog** | **chicken** | **frog** | **zebrafish** | **TFBS** | **score** | **% score** |
| un-weighted sequences | Motif 1 (u) | -78 to -64 | 6.30E-06 | 4.25E-09 | 4.25E-09 | 4.25E-09 | 4.25E-09 | 4.25E-09 | 1.73E-07 | 1.10E-06 | 2.36E-06 | 4.34E-06 | Pax4 | 22.94 | 76.46 |
|  | Motif 2 (u) | -752 to -738 | 3.20E-06 | 5.90E-09 | 9.98E-09 | 1.19E-09 | 1.19E-09 | 1.29E-08 | 1.47E-06 | 7.85E-07 | 1.05E-06 | 4.47E-07 | Pax4 | 22.87 | 76.22 |
|  | Motif 3 (u) | -1200 to -1186 | 5.60E-08 | 2.55E-07 | 1.76E-07 | 3.38E-10 | 3.38E-10 | 1.95E-08 | 8.33E-08 | 8.77E-07 | 1.53E-07 | 7.52E-09 | Pax4 | 23.11 | 77.04 |
|  | Motif 4 (u) | +2 to +16 | 1.90E-04 | 2.46E-08 | 3.21E-08 | 3.24E-09 | 1.00E-08 | 7.75E-09 | 3.21E-08 | 1.26E-06 | 1.18E-06 | 3.26E-06 | Pax4 | 23.27 | 77.55 |
|  | Motif 5 (u) | -431 to -417 | 5.20E-04 | 4.69E-09 | 4.69E-09 | 4.09E-07 | 8.30E-07 | 1.97E-07 | 8.61E-08 | 8.30E-07 | 2.91E-07 | 2.16E-07 | Pax5 | 24.61 | 82.03 |
|  | Motif 6 (u) | -305 to -291 | 2.50E-03 | 9.29E-09 | 1.52E-07 | 4.44E-08 | 2.23E-09 | 1.99E-08 | 2.23E-09 | 4.40E-06 | 2.66E-06 | 2.43E-06 | Pax5 | 23.04 | 76.81 |
|  | Motif 7 (u) | -1235 to -1222 | 8.10E-03 | 7.76E-08 | 7.62E-07 | 2.26E-07 | 1.09E-07 | 3.22E-06 | 5.43E-07 | 9.63E-07 | 1.46E-07 | 2.42E-07 | RXR::RAR_DR5 | 22.50 | 80.37 |
|  | Motif 8 (u) | -1298 to -1285 | 1.20E-03 | 1.20E-09 | 2.42E-08 | 2.29E-07 | 1.94E-07 | 1.83E-06 | 5.53E-06 | 1.23E-06 | 5.44E-08 | 4.43E-07 | Pax4 | 22.69 | 81.03 |
|  | Motif 9 (u) | -43 to -29 | 1.30E-02 | 4.73E-09 | 4.73E-09 | 1.74E-09 | 4.73E-09 | 1.74E-09 | 1.36E-06 | 4.22E-06 | 2.40E-06 | 6.70E-06 | Pax4 | 23.23 | 77.43 |
|  | Motif 10 (u) | -661 to -647 | 2.90E-03 | 9.39E-09 | 7.85E-08 | 1.78E-08 | 1.78E-08 | 7.05E-09 | 1.76E-09 | 7.70E-07 | 4.65E-06 | 8.54E-06 | REST | 24.77 | 82.56 |
| weighted sequences | *[Motif 3 (u)] |  | 8.20E-06 | 7.45E-07 | 5.60E-07 | 5.45E-10 | 5.45E-10 | 2.54E-08 | 1.23E-07 | 8.93E-07 | 1.35E-07 | 6.32E-09 |  |  |  |
|  | *[Motif 2 (u)] |  | 1.10E-03 | 3.59E-08 | 3.82E-08 | 1.26E-09 | 1.26E-09 | 1.28E-08 | 1.74E-06 | 4.24E-07 | 5.93E-07 | 3.57E-07 |  |  |  |
|  | Motif 11 (w) | -65 to -51 | 1.00E-03 | 3.86E-07 | 2.55E-08 | 3.02E-09 | 5.90E-09 | 5.90E-09 | 5.90E-09 | 1.19E-07 | 1.18E-06 | 1.73E-06 | Pax4 | 22.73 | 75.78 |
|  | *[Motif 5 (u)] |  | 3.00E-03 | 3.33E-09 | 4.47E-09 | 1.39E-06 | 2.23E-06 | 1.17E-07 | 7.57E-08 | 4.36E-07 | 1.07E-07 | 7.57E-08 |  |  |  |
|  | *[Motif 4 (u)] |  | 2.90E-02 | 2.98E-06 | 1.34E-07 | 4.01E-09 | 1.94E-08 | 8.08E-09 | 5.31E-08 | 7.01E-07 | 5.51E-07 | 2.19E-06 |  |  |  |
|  | Motif 12 (w) | -1234 to -1220 | 2.40E-04 | 2.98E-06 | 6.95E-07 | 9.67E-07 | 1.25E-07 | 5.83E-09 | 9.25E-10 | 8.24E-07 | 1.78E-08 | 5.14E-08 | Pax4 | 23.62 | 78.75 |
|  | Motif 13 (w) | -318 to -308 | 5.00E-01 | 4.31E-06 | 4.31E-06 | 6.46E-07 | 6.46E-07 | 6.46E-07 | 6.46E-07 | 2.02E-06 | 6.12E-06 | 9.26E-06 | INSM1 | 17.69 | 80.43 |
|  | Motif 14 (w) | -661 to -653 | 6.50E+00 | 2.37E-05 | 8.82E-06 | 2.95E-06 | 2.95E-06 | 1.87E-05 | 2.95E-06 | 8.82E-06 | 2.10E-05 | 8.82E-06 | REST | 15.53 | 86.30 |
|  | Motif 15 (w) | -300 to -290 | 2.10E+00 | 6.09E-06 | 8.22E-06 | 2.01E-07 | 2.01E-07 | 1.55E-06 | 2.01E-07 | 5.68E-06 | 6.75E-06 | 3.89E-06 | Pax5 | 17.54 | 79.71 |
|  | Motif 16 (w) | -1321 to -1311 | 5.60E-01 | 2.44E-06 | 3.47E-07 | 3.59E-06 | 2.27E-05 | 1.07E-06 | 4.84E-06 | 2.76E-06 | 1.81E-07 | 2.24E-06 | IRF1/IRF2 | 17.98 | 81.74 |

[w = weighted]

[u = un-weighted]

*same motif found in run with un-weighted sequences

^1^position relative to the translation start site codon ATG of human Ngb

^2^Average p-value un-weighted sequences: 8.48E-07

^3^Average p-value weighted sequences: 2.51E-06

Table S2b: Comprehensive results of the MEME analysis using shuffled sequences.

|  | **Motif** | **e-value** | **p-value^2,3^** | | | | | | | | | **Jaspar** | | |
| --- | --- | --- | --- | --- | --- | --- | --- | --- | --- | --- | --- | --- | --- | --- |
|  |  |  | **rat** | **mouse** | **rhesus** | **human** | **cow** | **dog** | **chicken** | **frog** | **zebrafish** | **TFBS** | **score** | **% score** |
| un-weighted sequences | Motif 1 (u) | 4.50E+04 | 2.81E-05 | 2.81E-05 | 3.84E-04 | 6.27E-05 | 6.27E-05 | 1.93E-04 | 1.23E-04 | 2.81E-05 | 2.81E-05 | GATA | 12.37 | 88.39 |
|  | Motif 2 (u) | 2.00E+04 | 7.46E-07 | 6.84E-07 | 7.14E-06 | 3.39E-07 | 3.39E-07 | 5.86E-06 | 1.15E-06 | 2.09E-09 | 1.24E-05 | Pax4 | 25.08 | 83.59 |
|  | Motif 3 (u) | 5.00E+04 | 1.57E-05 | 1.16E-04 | 3.03E-05 | 5.67E-05 | 1.16E-04 | 1.57E-05 | 4.40E-05 | 3.03E-05 | 7.13E-05 | Pax5 | 12.95 | 80.94 |
|  | Motif 4 (u) | 1.60E+05 | 6.11E-07 | 1.23E-05 | 1.35E-05 | 1.49E-05 | 2.81E-06 | 5.19E-06 | 2.57E-05 | 2.77E-05 | 2.34E-06 | znf143 | 18.18 | 82.64 |
|  | Motif 5 (u) | 2.40E+05 | 2.13E-04 | 2.13E-04 | 2.13E-04 | 2.13E-04 | 2.13E-04 | 3.96E-04 | 2.13E-04 | 3.96E-04 | 2.13E-04 | NFYA | 10.60 | 88.35 |
|  | Motif 6 (u) | 6.90E+04 | 1.79E-05 | 7.56E-07 | 8.20E-06 | 3.74E-07 | 1.19E-08 | 4.70E-08 | 3.55E-06 | 4.78E-07 | 3.04E-06 | Pax4 | 25.56 | 85.21 |
|  | Motif 7 (u) | 4.20E+05 | 1.26E-05 | 1.26E-05 | 6.11E-05 | 1.39E-04 | 7.00E-05 | 1.53E-04 | 3.82E-05 | 1.26E-05 | 4.84E-05 | HOXA5 | 12.53 | 78.32 |
|  | Motif 8 (u) | 1.10E+06 | 3.09E-05 | 1.05E-04 | 8.54E-05 | 5.72E-05 | 5.72E-05 | 1.18E-05 | 6.75E-05 | 1.05E-04 | 3.09E-05 | Sox17 | 15.16 | 94.73 |
|  | Motif 9 (u) | 1.80E+05 | 4.03E-05 | 1.46E-04 | 2.76E-05 | 2.76E-05 | 2.76E-05 | 8.65E-05 | 7.19E-05 | 1.12E-04 | 2.76E-05 | FOXI1 | 12.70 | 79.41 |
|  | Motif 10 (u) | 1.50E+05 | 2.59E-05 | 1.53E-06 | 3.49E-06 | 1.01E-05 | 2.04E-07 | 4.52E-05 | 8.65E-06 | 1.01E-05 | 3.91E-05 | Tal1::Gata1 | 17.71 | 80.52 |
| weighted sequences | Motif 1 (w) | 1.90E+04 | 1.36E-05 | 1.60E-04 | 3.93E-05 | 2.99E-04 | 1.36E-05 | 5.52E-05 | 1.36E-05 | 1.36E-05 | 2.70E-05 | RREB1 | 14.75 | 92.21 |
|  | Motif 2 (w) | 1.40E+06 | 4.10E-05 | 1.35E-04 | 4.10E-05 | 1.35E-04 | 1.35E-04 | 6.21E-05 | 1.91E-05 | 1.91E-05 | 7.72E-05 | RXRA::VDR | 13.21 | 82.56 |
|  | Motif 3 (w) | 1.50E+06 | 1.23E-04 | 4.52E-05 | 8.19E-05 | 2.05E-04 | 1.19E-05 | 5.84E-05 | 2.23E-05 | 4.52E-05 | 3.16E-05 | PPARG::RXRA | 13.35 | 83.45 |
|  | Motif 4 (w) | 4.00E+05 | 2.05E-04 | 2.95E-05 | 3.25E-04 | 2.95E-05 | 1.59E-05 | 7.03E-05 | 1.59E-05 | 2.95E-05 | 4.41E-05 | Ar | 13.96 | 87.22 |
|  | Motif 5 (w) | 2.60E+06 | 2.80E-04 | 2.80E-04 | 6.36E-04 | 6.36E-04 | 2.80E-04 | 2.80E-04 | 2.80E-04 | 2.80E-04 | 2.80E-04 | HNF1A | 10.22 | 85.18 |
|  | Motif 6 (w) | 6.40E+06 | 2.58E-04 | 2.58E-04 | 2.58E-04 | 5.12E-04 | 2.58E-04 | 2.58E-04 | 2.58E-04 | 2.58E-04 | 5.12E-04 | Arnt::Ahr | 11.46 | 95.53 |
|  | Motif 7 (w) | 1.60E+06 | 1.46E-04 | 1.46E-04 | 9.49E-05 | 1.11E-04 | 7.90E-05 | 3.66E-05 | 1.61E-05 | 4.93E-05 | 1.61E-05 | Ar | 13.13 | 82.07 |
|  | Motif 8 (w) | 1.40E+06 | 2.07E-04 | 4.07E-05 | 4.07E-05 | 1.36E-05 | 1.08E-04 | 4.07E-05 | 1.36E-05 | 9.41E-05 | 9.41E-05 | HLF | 13.49 | 84.34 |
|  | Motif 9 (w) | 2.50E+05 | 1.10E-04 | 6.22E-05 | 2.39E-04 | 1.56E-05 | 3.09E-05 | 7.95E-05 | 4.82E-05 | 1.56E-05 | 3.09E-05 | znf143 | 13.11 | 81.95 |
|  | Motif 10 (w) | 2.70E+06 | 1.72E-04 | 8.78E-05 | 1.61E-04 | 8.78E-05 | 2.09E-04 | 3.10E-05 | 1.09E-05 | 1.09E-05 | 2.10E-05 | RUNX1 | 13.67 | 85.45 |

[w = weighted]

[u = un-weighted]

^2^Average p-value un-weighted sequences: 6.33E-05

^3^Average p-value weighted sequences: 1.28E-04
