# Supplementary material for: Comparative Genomics of Neuroglobin Reveals Its Early Origins
Source: PLoS One. 2012 Oct 25;7(10):e47972. doi: 10.1371/journal.pone.0047972 (PMC3485006; doi:10.1371/journal.pone.0047972)
Supplement: Table S3 — Comprehensive results of the FootPrinter analysis. For each motif the parsimony score, the start and end positions of the sites relative to the translation start codon ATG and the results from the comparison against the Jaspar database are given. (DOC) [file pone.0047972.s007.doc]

Table S3: Comprehensive results of the CONREAL analysis.

|  | **specie** | **position^1^** | **strand** | **score** | **rel. score (%)** |
| --- | --- | --- | --- | --- | --- |
| **Churchill (M00986)** | Chicken | -143 to -138 | 1 | 7.901 | 1 |
|  | Chicken | -129 to -124 | 1 | 4.722 | 0.85 |
|  | Chicken | -125 to -120 | 1 | 2.544 | 0.75 |
|  | Chicken | -118 to -113 | 1 | 6.205 | 0.92 |
|  | Cow | -133 to -128 | 1 | 7.524 | 0.98 |
|  | Dog | -128 to -123 | 1 | 5.706 | 0.9 |
|  | Dog | -124 to -119 | 2 | 6.604 | 0.94 |
|  | Dog | -108 to -103 | 1 | 6.205 | 0.92 |
|  | Dog | -104 to -99 | 1 | 2.544 | 0.75 |
|  | Dog | -100 to -95 | 1 | 5.53 | 0.89 |
|  | Frog | -125 to -120 | 2 | 4.88 | 0.86 |
|  | Frog | -120 to -115 | 1 | 4.346 | 0.84 |
|  | Frog | -103 to -98 | 1 | 2.527 | 0.75 |
|  | Human | -136 to -131 | 2 | 7.901 | 1 |
|  | Human | -116 to -111 | 1 | 6.418 | 0.93 |
|  | Mouse | -138 to -133 | 2 | 5.53 | 0.89 |
|  | Mouse | -134 to -129 | 2 | 5.807 | 0.9 |
|  | Mouse | -118 to -113 | 1 | 3.834 | 0.81 |
|  | Mouse | -109 to -104 | 1 | 6.177 | 0.92 |
|  | Mouse | -102 to -97 | 2 | 6.009 | 0.91 |
|  | Rat | -138 to -133 | 2 | 6.205 | 0.92 |
|  | Rat | -122 to -117 | 1 | 3.834 | 0.81 |
|  | Rat | -113 to -108 | 1 | 6.177 | 0.92 |
|  | Rat | -105 to -100 | 2 | 6.042 | 0.91 |
|  | Rhesus monkey | -133 to -128 | 1 | 7.901 | 1 |
|  | Rhesus monkey | -108 to -103 | 1 | 4.722 | 0.85 |
|  | Rhesus monkey | -101 to -96 | 1 | 5.289 | 0.88 |
|  | Zebrafish | -142 to -137 | 1 | 4.223 | 0.83 |
| **CdxA (M00101)** | Chicken | -1361 to -1355 | 2 | 2.213 | 0.78 |
|  | Chicken | -1349 to -1343 | 2 | 3.148 | 0.82 |
|  | Chicken | -1339 to -1333 | 1 | 2.548 | 0.8 |
|  | Chicken | -1211 to -1205 | 2 | 1.884 | 0.77 |
|  | Cow | -1349 to -1343 | 1 | 2.323 | 0.79 |
|  | Cow | -1229 to -1223 | 1 | 4.351 | 0.87 |
|  | Cow | -1222 to -1216 | 1 | 2.837 | 0.81 |
|  | Cow | -1207 to -1201 | 1 | 4.499 | 0.88 |
|  | Dog | -1328 to -1322 | 1 | 4.507 | 0.88 |
|  | Dog | -1321 to -1315 | 1 | 1.457 | 0.75 |
|  | Dog | -1250 to -1244 | 1 | 4.339 | 0.87 |
|  | Frog | -1364 to -1358 | 2 | 3.365 | 0.83 |
|  | Frog | -1359 to -1353 | 2 | 4.579 | 0.88 |
|  | Frog | -1337 to -1331 | 2 | 2.006 | 0.78 |
|  | Frog | -1323 to -1317 | 1 | 3.143 | 0.82 |
|  | Frog | -1238 to -1232 | 2 | 2.572 | 0.8 |
|  | Frog | -1231 to -1225 | 1 | 3.265 | 0.83 |
|  | Frog | -1215 to -1209 | 2 | 4.886 | 0.89 |
|  | Frog | -1210 to -1204 | 2 | 3.076 | 0.82 |
|  | Human | -1320 to -1314 | 1 | 4.507 | 0.88 |
|  | Human | -1247 to -1241 | 1 | 4.431 | 0.88 |
|  | Human | -1240 to -1234 | 1 | 4.42 | 0.88 |
|  | Human | -1232 to -1226 | 1 | 3.143 | 0.82 |
|  | Human | -1218 to -1212 | 2 | 4.502 | 0.88 |
|  | Human | -1211 to -1205 | 2 | 4.286 | 0.87 |
|  | Mouse | -1359 to -1353 | 1 | 1.375 | 0.75 |
|  | Mouse | -1354 to -1348 | 2 | 2.082 | 0.78 |
|  | Mouse | -1343 to -1337 | 2 | 2.44 | 0.79 |
|  | Mouse | -1250 to -1244 | 2 | 6.844 | 0.98 |
|  | Rat | -1355 to -1349 | 1 | 3.076 | 0.82 |
|  | Rat | -1329 to -1323 | 2 | 1.613 | 0.76 |
|  | Rat | -1244 to -1238 | 2 | 3.116 | 0.82 |
|  | Rhesus monkey | -1362 to -1356 | 1 | 2.596 | 0.8 |
|  | Rhesus monkey | -1226 to -1220 | 1 | 1.623 | 0.76 |
|  | Zebrafish | -1359 to -1353 | 2 | 3.664 | 0.84 |
|  | Zebrafish | -1343 to -1337 | 1 | 2.991 | 0.82 |
|  | Zebrafish | -1221 to -1215 | 1 | 2.023 | 0.78 |
| **MZF_1-4 (MA0056)** | Chicken | -143 to -138 | 1 | 5.893 | 0.85 |
|  | Chicken | -127 to -122 | 1 | 4.156 | 0.78 |
|  | Chicken | -87 to -82 | 2 | 4.34 | 0.78 |
|  | Cow | -133 to -128 | 1 | 8.51 | 0.97 |
|  | Cow | -86 to -81 | 2 | 4.34 | 0.78 |
|  | Dog | -102 to -97 | 1 | 5.893 | 0.85 |
|  | Dog | -90 to -85 | 1 | 4.156 | 0.78 |
|  | Frog | -124 to -119 | 2 | 4.34 | 0.78 |
|  | Human | -136 to -131 | 2 | 5.893 | 0.85 |
|  | Human | -96 to -91 | 1 | 7.939 | 0.95 |
|  | Mouse | -138 to -133 | 2 | 4.989 | 0.81 |
|  | Mouse | -101 to -96 | 2 | 8.252 | 0.96 |
|  | Mouse | -88 to -83 | 1 | 5.893 | 0.85 |
|  | Rat | -113 to -108 | 1 | 5.322 | 0.83 |
|  | Rat | -105 to -100 | 2 | 8.252 | 0.96 |
|  | Rat | -92 to -87 | 1 | 5.893 | 0.85 |
|  | Rhesus monkey | -133 to -128 | 1 | 5.893 | 0.85 |
|  | Rhesus monkey | -106 to -101 | 1 | 4.156 | 0.78 |
|  | Rhesus monkey | -86 to -81 | 1 | 3.769 | 0.76 |
|  | Zebrafish | -142 to -137 | 1 | 4.156 | 0.78 |
|  | Zebrafish | -95 to -90 | 1 | 4.846 | 0.81 |
| **AP2alpha (M00469)** | Chicken | -147 to -139 | 1 | 3.917 | 0.79 |
|  | Chicken | -138 to -130 | 1 | 8.893 | 0.95 |
|  | Chicken | -127 to -119 | 1 | 3.739 | 0.78 |
|  | Cow | -143 to -135 | 2 | 6.836 | 0.88 |
|  | Cow | -115 to -107 | 2 | 5.598 | 0.84 |
|  | Cow | -108 to -100 | 1 | 5.422 | 0.84 |
|  | Dog | -135 to -127 | 1 | 6.676 | 0.88 |
|  | Dog | -106 to -98 | 1 | 3.917 | 0.79 |
|  | Frog | -125 to -117 | 1 | 2.855 | 0.75 |
|  | Frog | -108 to -100 | 1 | 2.855 | 0.75 |
|  | Human | -127 to -119 | 2 | 6.265 | 0.87 |
|  | Mouse | -122 to -114 | 2 | 6.138 | 0.86 |
|  | Mouse | -115 to -107 | 1 | 9.058 | 0.96 |
|  | Rat | -133 to -125 | 2 | 6.091 | 0.86 |
|  | Rat | -126 to -118 | 2 | 6.138 | 0.86 |
|  | Rat | -119 to -111 | 1 | 9.058 | 0.96 |
|  | Rhesus monkey | -143 to -135 | 2 | 6.265 | 0.87 |
|  | Rhesus monkey | -106 to -98 | 1 | 3.739 | 0.78 |
|  | Zebrafish | -147 to -139 | 1 | 3.049 | 0.76 |

^1^position relative to the translation start codon ATG
