# Supplementary material for: Comparative Genomics of Neuroglobin Reveals Its Early Origins
Source: PLoS One. 2012 Oct 25;7(10):e47972. doi: 10.1371/journal.pone.0047972 (PMC3485006; doi:10.1371/journal.pone.0047972)
Supplement: Table S4 — Comprehensive results of the CONREAL analysis. The score, relative score, strand, start and end position relative to the translation start codon ATG for each binding site are given. (DOC) [file pone.0047972.s008.doc]

Table S4: Comprehensive results of the FootPrinter analysis.

| **Motif** | **position^1^** | **Parsimony score** | **Jaspar** | | |
| --- | --- | --- | --- | --- | --- |
|  |  |  | **TFBS** | **score** | **% score** |
| Motif 1* | -325 to -318 -319 to -312 | 3.00 | MZF1 5-13 | 14.26 | 89.15 |
| Motif 2* |  | 3.00 | SP1 | 13.83 | 86.45 |
| Motif 3 | -318 to -311 -227 to -220 | 3.00 | Klf4 | 12.84 | 91.75 |
| Motif 4 | -1341 to -1334 | 3.00 | Zfx | 13.37 | 83.57 |
| Motif 5* | -428 to -421 -786 to -779 | 3.00 | Tal1::Gata1 | 12.94 | 80.85 |
| Motif 6* |  | 3.00 | Tcfcp2l1 | 13.21 | 82.55 |
| Motif 7 | -1179 to -1172 -249 to -242 -242 to -235 | 3.00 | CTCF | 13.52 | 84.48 |

^1^position relative to the translation start site codon ATG of human Ngb

*Motifs 1+2 and motifs 5+6 correspond to same positions in the human Ngb promoter regions, but motifs are based on different positions in other vertebrates. Thus, the derived position weight matrixes and results of the comparison against the Jaspar database differ.
